# Colorectal Cancer Outcomes of Robotic Surgery Using the Hugo™ RAS System: The First Worldwide Comparative Study of Robotic Surgery and Laparoscopy

**DOI:** 10.3390/cancers17071164

**Published:** 2025-03-30

**Authors:** Giacomo Calini, Stefano Cardelli, Ioana Diana Alexa, Francesca Andreotti, Michele Giorgini, Nicola Maria Greco, Fiorella Agama, Alice Gori, Dajana Cuicchi, Gilberto Poggioli, Matteo Rottoli

**Affiliations:** 1 Surgery of the Alimentary Tract, IRCCS Azienda Ospedaliero-Universitaria di Bologna, 40139 Bologna, Italy; 2 Department of Medical and Surgical Sciences, Alma Mater Studiorum-University of Bologna, 40139 Bologna, Italy

**Keywords:** colorectal cancer, robotic surgery, Medtronic Hugo-RAS, laparoscopic, comparative study

## Abstract

**Background/Objectives**: The aim of the study was to compare the perioperative and oncologic outcomes of patients who underwent surgery for colorectal cancer (CRC) performed using laparoscopy or using the Medtronic Hugo™ Robotic-Assisted Surgery (RAS) system. **Methods:** This is a retrospective comparative single-center study of consecutive minimally invasive surgeries for CRC performed by two colorectal surgeons with extensive laparoscopic experience at the beginning of their robotic expertise. Patients were not selected for the surgical approach, but waiting lists and operating room availability determined whether the patients were in the robotic group or the laparoscopic group. The primary outcome was to compare 30-day postoperative complications according to the Clavien–Dindo classification and the Complication Comprehensive Index (CCI). The secondary outcomes included operating times, conversion rates, intraoperative complications, length of hospital stays (LOS), readmission rates, and short-term oncologic outcomes, such as the R0 resection, the number of lymph nodes harvested, the total mesorectal excision (TME) quality, and the circumferential resection margin (CRM). **Results:** Of the 109 patients, 52 underwent robotic and 57 laparoscopic CRC surgery. Patient demographic and clinical characteristics were similar in the two groups. There was no significant difference between the robotic and the laparoscopic groups regarding postoperative complications, the Clavien–Dindo classification, and the CCI. They also had similar operating times, conversion rates, intraoperative complications, LOSs, readmission rates, and short-term oncologic outcomes (the lymph nodes harvested, the R0 resection, TME quality, and CRM status). **Conclusions:** This study reports the largest cohort of CRC surgery performed using the Medtronic Hugo™ RAS system and is the first comparative study with laparoscopy. The perioperative and oncologic outcomes were similar, demonstrating that the Medtronic Hugo™ RAS system is safe and feasible for CRC as compared to laparoscopic surgery, even at the beginning of the robotic experience.

## Simple Summary

Colorectal cancer (CRC) is a leading cause of cancer-related illness and death worldwide. Surgery is the main treatment for CRC, and minimally invasive techniques are preferred because they offer similar oncologic outcomes to open surgery, with less pain, faster recovery, and shorter hospital stays. Robotic-assisted surgery (RAS), like the Medtronic Hugo™ system, helps improve surgical precision. This study compared CRC surgeries performed with the Hugo™ RAS system and traditional laparoscopy by two expert laparoscopic surgeons at the beginning of their robotic experience. A total of 109 patients (52 RAS and 57 laparoscopic) were analyzed, with the type of surgical approach chosen based on operating room availability. The results showed no significant differences between the two methods regarding complications, surgery time, hospital stay, or cancer outcomes. Both methods performed equally well regarding tumor removal and lymph node count. The study concluded that robotic surgery with the Hugo™ RAS system is as safe and effective as laparoscopic surgery, even for surgeons new to RAS.

## 1. Introduction

Colorectal cancer (CRC) is a leading cause of cancer-related illness and death worldwide [1]. Surgical resection remains the cornerstone of treatment for localized disease [2,3]. Current guidelines endorse minimally invasive techniques since these approaches provide oncologic outcomes comparable to open surgery while offering reduced postoperative pain, faster recovery, and shorter hospital stays [2,4]. Recent advancements in minimally invasive surgery have introduced robotic-assisted surgery (RAS) to enhance surgical precision, improve dexterity, and reduce complications [5,6].

The Medtronic Hugo™ RAS system stands out as a novel platform owing to its modular and flexible design, open console, and cost-effectiveness [7]. It can expand access to robotic surgery for laparoscopic surgeons and improve patient outcomes by means of precise tumor resection, reduced blood loss, and faster recovery times [8]. A few consecutive series of CRC resections performed with the Hugo™ RAS system have been published [9,10,11]. However, no comparative study with laparoscopy has been carried out.

The aim of this study was to report the largest cohort of consecutive CRC surgeries performed with the Medtronic Hugo™ RAS system and compare the outcomes with those of laparoscopic surgery performed by the same surgeons.

## 2. Materials and Methods

### 2.1. Study Design and Settings

This retrospective single-center study enrolled consecutive adult patients who underwent minimally invasive surgery for CRC (laparoscopic or robotic) at the authors’ institution between April 2023 and October 2024. All the surgeries were performed by a non-robotic surgical team at the study’s onset. The same two main operators performed all the surgeries (both laparoscopic and robotic) during the same period of time. They had extensive experience in laparoscopic colorectal procedures (>500 cases) but were naïve to robotic surgery at the study’s onset. This methodology was selected to reduce biases for inter-surgeon and temporal variability. All the robotic surgeries were performed using the Medtronic Hugo™ RAS platform (Medtronic, Minneapolis, MN, USA); both surgeons completed the platform certification. The consecutive series started in April 2023 at the beginning of the authors’ institutional robotic program. In their institution, CRC cases are managed via a multidisciplinary colorectal pathway involving the institution’s surgeons, oncologists, radiologists, radiotherapists, and dedicated nurses. The patients were not intentionally selected to undergo robotic surgery over laparoscopy; factors, such as operating room availability and waiting lists, primarily determined the approach. Patients under 18 years of age, with any histologic diagnosis other than colorectal adenocarcinoma, those undergoing emergent surgery, and those with incidental findings of CRC were excluded from the analysis.

### 2.2. Study Aims

The primary outcome was to compare the 30-day overall postoperative complication rate according to the Clavien–Dindo classification [12] and the Complication Comprehensive Index (CCI) [13]. The secondary outcomes included comparing operating times, conversion rates, intraoperative complications, length of hospital stays (LOS), readmission rates, and short-term oncologic outcomes, such as the R0 resection, the number of lymph nodes harvested, the total mesorectal excision (TME) quality, and the circumferential resection margin (CRM).

### 2.3. Variables and Data Presentation

The variables of interest were reviewed retrospectively from a prospectively-maintained database and included demographics, tumor characteristics, neoadjuvant treatment, and procedure-related data. The patients’ comorbidity statuses were classified using the American Society of Anesthesiologists (ASA) score and the Charlson Comorbidity Index [14]. Preoperative clinical and pathological stages were characterized according to the eighth edition of the American Joint Committee Cancer Staging Manual [15]. The multidisciplinary teams determined the neoadjuvant therapy regimens or the first-line chemotherapy regimen for stage IV colon cancer based on the evidence available and individual patient characteristics. The operating details collected included the type of surgical procedure performed; the operating time; the anastomosis type (intracorporeal vs. extracorporeal); the creation of a stoma; intraoperative complications (including the type, management, and clinical consequences); and conversion to open surgery (defined as an unplanned laparotomy created for any purpose other than specimen extraction) [16]. The postoperative outcomes included the length of hospital stays; 30-day complications (e.g., ileus, urinary retention, wound infection, abdominal collection, deep vein thrombosis/pulmonary embolism, pneumonia, anastomotic leak); readmission rates; reoperation rates; and 30-day mortalities. Postoperative complications were classified according to the Clavien–Dindo classification system and measured with the CCI [12,13]. Anastomotic leakage was defined using the International Study Group of Rectal Cancer (ISREC) criteria, including pelvic abscesses near the anastomosis [17]. The oncologic surrogate outcomes collected included the number of lymph nodes harvested, the disease prognostic stage based on pathologic Tumor, Node and Metastasis (pTNM) staging, and R0 resection (microscopically margin-negative resection). The proctectomy-related specific characteristics collected were TME quality (categorized as complete, nearly complete, or incomplete) and CRM positivity (defined as a tumor less than 1 mm from the inked non-peritoneal surface). The study was conducted according to the Guidelines for Strengthening the Reporting of Observational Studies in Epidemiology (STROBE, Table S1) [18].

### 2.4. The Perioperative Setting, Surgical Technique, and Robotic Setup

Preoperative preparation with oral antibiotics plus mechanical bowel preparation was administered in most cases [19]. In a few cases, they received only mechanical bowel preparation for antibiotic allergy or oral antibiotics for sub-occlusive symptoms. Intraoperative fluids required anesthesia case planning (complexity and duration) to ensure adequate total fluid volume, and early postoperative oral intake was the standard pathway according to our enhanced recovery pathway [20,21]. Intracorporeal or transanal anastomoses are the preferred techniques to reestablish intestinal continuity, ensuring the best surgical outcomes [22,23].

All the surgical procedures reported in this study were surgical oncological resections performed with curative intent and according to the oncological principles of central ligation, mesocolon/mesorectal excision, and distance from cancer to resection margin. In both the RAS and laparoscopic approaches, central ligation was achieved through a laparoscopic/assistant port with Hem-o-lok clips or the Signia Tri-Stapling System.

In right and left colectomies, the mesentery mobilization was preferentially performed medial to lateral, and mesenteric dissection was mainly accomplished with bipolar and energy devices (e.g., LigaSure vessel sealing or Thunderbeat) according to surgeon preferences and patient characteristics. When the mesentery was particularly thickened, its division was made through a multiple overlapping burn technique—a combination of bipolar energy and energy devices similar to the technique proposed for Crohn’s Disease [24]. In proctectomies, the TME was performed mainly with Thunderbeat in the laparoscopic approach while in RAS, it was performed with a robotic monopolar scissor and bipolar grasper. A Signia Tri-Stapling System was used to achieve bowel resections.

After the oncological resections, intestinal continuity was reestablished according to the type of colorectal resection. After a right colectomy, an intracorporeal anastomosis configuration was performed in most cases, with a side-to-side intracorporeal anastomosis with a Signia Tri-Stapling System and a V-Loc barbed suture for enterotomy closure. For a left colectomy or a proctectomy, a transanal termino-terminal colorectal anastomosis (Knight–Griffen) was performed. A tailored robotic setup was used according to the already published article for left colectomies and proctectomies. This setup grants the platform exceptional flexibility in multi-quadrant abdominal procedures, requiring minimal adjustments between dockings to optimize the machine’s performance [25]. Right colectomies were performed with the standard robotic setup configuration.

### 2.5. Statistical Analysis

All the statistical analyses were carried out using R version 4.3.2 [26]. The continuous variables are reported as medians [interquartile range (IQR)], while categorical variables are reported as frequencies (percentages). The differences in the continuous variables between the robotic and the laparoscopic groups were assessed using the Wilcoxon rank-sum test. For the categorical variables, the comparisons were carried out using the Chi-squared or Fisher’s exact test, depending on the frequencies expected. A two-sided *p*-value of <0.05 was considered statistically significant.

## 3. Results

A total of 109 patients operated on by the same two surgeons underwent minimally invasive surgery for CRC (52 robotic and 57 laparoscopic).

The demographic and clinical characteristics of the patients in the two groups were similar (Table 1) for age, sex, smoking, body mass index (BMI), previous abdominal surgery, cancer stage, and comorbidities as assessed by the ASA and the Charlson comorbidity index. Notably, the robotic group had a lower rate of patients receiving preoperative chemoradiotherapy, with a similar rate of stage IV colon cancer, which was resectable after first-line therapy.

The surgical details and outcomes are reported in Table 2. A total of 41 patients (79%) in the robotic group and 38 (67%) in the laparoscopic group underwent a colon cancer resection. There were no significant differences between the robotic and the laparoscopic groups regarding operating times, conversion rates, intraoperative complications, and stoma creations.

Postoperative complications (Table 2), evaluated according to Clavien–Dindo grade and the CCI, did not significantly differ between the robotic and the laparoscopic groups. Moreover, the LOSs were similar, with no readmissions in the robotic group and only three readmissions in the laparoscopic group (two subacute bowel obstructions treated conservatively, and one anastomotic leak which underwent transanal revision).

Pathological assessment of the oncologic outcomes is reported in Table 3. Oncologic outcomes, including the number of lymph nodes harvested, R0 resection, and pTNM, were comparable between the two groups. The TME quality and CRM involvement in rectal cancer did not differ significantly.

## 4. Discussion

This study reports the largest cohort of consecutive CRC surgeries performed using the Medtronic Hugo™ RAS system as compared with laparoscopy. The perioperative and oncologic outcomes were similar for the robotic and the laparoscopic procedures performed. The procedures were performed during the same period by the same two robotic-naïve and laparoscopically-expert surgeons. This was proof of the safety and feasibility of an expert laparoscopic colorectal surgeon shifting to performing CRC surgery using the Medtronic Hugo™ RAS system without impacting patient outcomes.

Notably, the operating time was 10 min longer in the robotic group. However, this was not statistically significant. This parallels previous publications and might indicate a potential benefit of the Hugo RAS platform, which requires additional investigation [25,27]. Regarding the oncologic outcomes, robotic and laparoscopy lymph node retrievals, TME qualities, CRM positivity rates, and adequate timings of adjuvant chemotherapy were not statistically different; however, they were slightly better for the robotic surgery. This finding supports earlier research indicating that robotic surgery does not compromise oncologic safety and might offer technical advantages for precise dissection, particularly in narrow pelvic spaces [28,29,30].

While earlier studies have demonstrated the safety and feasibility of other RAS systems in the surgical management of colorectal cancer, primarily the Da Vinci platform [31], data regarding the use of the Hugo™ RAS system are limited [9,10,11]. To the best of the authors’ knowledge, this was the largest cohort of CRC patients operated on using the Hugo RAS platform, and is the first report to compare the postoperative complications and oncologic outcomes of this platform with laparoscopy. Of the robotic platforms available, the Medtronic Hugo™ RAS platform represents a novel alternative, designed to offer flexibility and adaptability. Its key features include an open console which facilitates communication within the operating room, and supports training and a modular setup with four independent robotic arm carts, enabling custom docking configurations [8,25]. This flexibility is particularly valuable in terms of multi-quadrant access and the tailored surgical approaches required for CRC surgery, as shown by previous reports on colorectal surgery with the Hugo™ RAS system [32,33].

Since surgical resection remains the cornerstone of treatment for nonmetastatic disease, much effort has been made to find the optimal approach for achieving the best clinical and oncological outcomes. Current guidelines endorse the use of minimally invasive techniques, especially laparoscopic surgery, since these approaches provide oncologic outcomes comparable to open surgery while offering additional advantages, such as reduced postoperative pain, faster recovery, and shorter hospital stays [2,34]. However, laparoscopy also presents significant challenges in CRC surgery, including restricted instrument mobility and limited visualization, specifically in the deep pelvis during rectal cancer procedures requiring TME [28,29,30].

The use of RAS in colorectal cancer surgery has expanded significantly in recent years with encouraging results in terms of safety, feasibility, and patient outcomes [5,6]. The present study supports the adoption of the Medtronic Hugo™ RAS platform for robotic-naïve and laparoscopically expert surgeons as has been demonstrated in the past for other benign colorectal procedures [27]. The authors believe that the choice between robotic and laparoscopic surgery should consider institutional resources, surgeon expertise, and patient-specific factors rather than assuming the clinical superiority of one technique over the other.

A significant strength of this study is its real-world clinical setting, which involved a consecutive non-selected cohort of patients undergoing minimally invasive CRC surgery by the same two surgeons (performing both robotic and laparoscopic cases). However, several limitations must be acknowledged: the single-center retrospective design requires additional research to allow for generalizability; the sample size restricts the ability to detect slight differences between the two techniques; the surgeons’ initial experience with RAS at the study’s onset may have influenced operative efficiency; the data do not allow for an analysis of each techniques cost-effectiveness; and short-term follow-up is a surrogate for long-term oncologic outcome.

Additional multicenter studies with larger sample sizes and longer follow-up periods are warranted to better assess the long-term oncologic efficacy, ergonomics, and cost-effectiveness of robotic-assisted CRC surgery with the Medtronic Hugo™ RAS platform. Future studies should also explore patient-reported outcomes, the progression of the surgeon’s learning curve, and specific subgroups which may benefit more from robotic techniques, such as low rectal cancer cases requiring precise mesorectal excision.

## 5. Conclusions

This study demonstrates that the Medtronic Hugo™ RAS platform is safe and feasible for CRC surgery as compared with laparoscopic surgery; it has similar perioperative and oncologic outcomes, even at the beginning of the robotic experience.

## Figures and Tables

**Table 1:** Baseline Characteristics.

Variables	Robotic (N = 52)	Laparoscopic (N = 57)	Total (N = 109)	*p*-Value
**Age, year**	67 (58–77)	68 (60–76)	109	0.74
**Male gender**	28 (54%)	31 (54%)	109	1
**BMI, kg/mq**	26 (23–29)	25 (22–30)	109	0.57
**Smoking history**	51	53	104	0.68
**Never smoked**	27 (53%)	25 (47%)		
**Suspended**	17 (33%)	22 (42%)		
**Smoking now**	7 (14%)	6 (11%)		
**ASA score**	2 (2–3)	2 (2–3)	109	0.27
**1**	3 (6%)	3 (5%)		
**2**	26 (50%)	26 (46%)		
**3**	20 (38%)	28 (49%)		
**4**	3 (6%)	0		
**Previous abdominal surgeries**	23 (44%)	26 (46%)	109	1
**Charlson comorbidity**	5 (4–7)	5 (4–7)	109	0.88
**Cancer location**			109	0.43
**Right colon**	27 (52%)	22 (39%)		
**Transverse colon**	1 (2%)	1 (2%)		
**Left colon**	13 (25%)	15 (26%)		
**Rectum**	11 (21%)	19 (33%)		
**Preoperative chemotherapy**	2 (4%)	3 (5%)	109	0.44
**Preoperative chemoradiotherapy**	3 (6%)	14 (25%)	109	*0.004*
**Cancer prognostic stage**			109	0.41
**Stage 0**	3 (6%)	4 (7%)		
**Stage I**	22 (42%)	14 (25%)		
**Stage II**	15 (29%)	20 (35%)		
**Stage III**	8 (15%)	12 (21%)		
**Stage IV**	4 (8%)	7 (12%)		

Abbreviations: BMI: Body Mass Index, ASA: American Society of Anesthesiologists; Data are expressed as No. (%) or Median (1st quartile–3rd quartile).

**Table 2:** Surgical details and 30-day postoperative outcomes.

Variables	Robotic (N = 52)	Laparoscopic (N = 57)	Total (N = 109)	*p*-Value
**Duration of surgery**	225 (204–305)	214 (190–280)	109	0.11
**Surgical procedure**			109	0.28
**Right colectomy**	27 (52%)	22 (39%)		
**Left colectomy**	14 (27%)	16 (28%)		
**Proctectomy**	11 (21%)	19 (33%)		
Intracorporeal anastomosis *	26 (96%)	20 (91%)	49	0.58
**Stoma creation**			109	0.13
**None**	45 (88%)	41 (72%)		
**Colostomy**	3 (6%)	4 (7%)		
**Ileostomy**	4 (8%)	12 (21%)		
**Intraoperative complications**	3 (6%)	3 (5%)	109	0.88
**Open conversion**	3 (6%)	1 (2%)	109	0.34
**Postoperative complications CD III-IV**			109	0.46
**Ileus**	0	1 (2%)		
**Bleeding**	0	1 (2%)		
**Abdominal collection**	1 (2%)	0		
**Venous thromboembolism**	0	0		
**Anastomotic leak**	1 (2%)	2 (4%)		
**CCI**	0 (0–12)	0 (0–21)	109	0.29
**Length of stay, days**	7 (5–8)	7 (6–9)	109	0.35
**Readmission**	0	3 (5%)	109	0.25
**Adjuvant chemotherapy**			108	0.62
**No indications ^**	35 (67%)	35 (61%)		
**Started within 8 weeks**	16 (31%)	18 (32%)		
**Delayed or not started for postoperative complication**	1 (2%)	3 (5%)		

Abbreviations: CD: Clavien–Dindo, CCI: Complication Comprehensive Index. Data are expressed as No. (%) or Median (1st quartile–3rd quartile). * based on a total of 27 robotic and 22 laparoscopic ileocolic anastomoses. ^ according to tumor stage, patient comorbidity, and patient refusal.

**Table 3:** Pathological assessment of oncologic outcomes.

Variables	Robotic (N = 52)	Laparoscopic (N = 57)	Total (N = 109)	*p*-Value
**Harvested lymph nodes**	17 (14–22)	18 (13–24)	109	0.3
**R0 resection**	51 (98%)	54 (95%)	109	0.35
CRM positive *	1 (9%)	2 (11%)	30	1
TME *	11	19	30	0.46
**Complete**	10 (91%)	13 (69%)		
**Nearly complete**	1 (9%)	5 (26%)		
**Incomplete**	0	1 (5%)		
**Tumor stage**			109	0.2
**T0/Tx**	3 (6%)	6 (11%)		
**T1**	10 (19%)	7 (12%)		
**T2**	15 (29%)	8 (14%)		
**T3**	20 (28%)	28 (49%)		
**T4**	4 (8%)	8 (14%)		
**Nodal stage**			109	0.28
**N0**	41 (79%)	40 (70%)		
**N1**	11 (21%)	14 (25%)		
**N2**	0	3 (5%)		

Abbreviations: CRM: Circumferential Resection Margin, TME: Total Mesorectal Excision. Data are expressed as No. (%) or Median (1st quartile–3rd quartile). * based on a total of 11 robotic and 19 laparoscopic proctectomies.

## Data Availability

The data supporting the findings of this study are not openly available due to privacy and sensitivity reasons. Anonymized data can be made available from the corresponding author upon reasonable request. Data are located in controlled and secured access data storage at Alma Mater Studiorum—University of Bologna.

## Supplementary Materials

The following supporting information can be downloaded at: https://www.mdpi.com/article/10.3390/cancers17071164/s1, Table S1: STROBE Statement—checklist of items that should be included in reports of observational studies.
